# Overexpression of PLOD3 promotes tumor progression and poor prognosis in gliomas

**DOI:** 10.18632/oncotarget.24594

**Published:** 2018-02-28

**Authors:** Chia-Kuang Tsai, Li-Chun Huang, Wen-Chiuan Tsai, Shih-Ming Huang, Jiunn-Tay Lee, Dueng-Yuan Hueng

**Affiliations:** ^1^ Graduate Institute of Medical Sciences, National Defense Medical Center, Taipei, Taiwan, ROC; ^2^ Department of Neurology, Tri-Service General Hospital, National Defense Medical Center, Taipei, Taiwan, ROC; ^3^ Department of Biochemistry, National Defense Medical Center, Taipei, Taiwan, ROC; ^4^ Department of Pathology, Tri-Service General Hospital, National Defense Medical Center, Taipei, Taiwan, ROC; ^5^ Department of Neurological Surgery, Tri-Service General Hospital, National Defense Medical Center, Taipei, Taiwan, ROC; ^6^ Penghu Branch of Tri-Service General Hospital, Penghu, Taiwan, ROC

**Keywords:** PLOD3, gene expression omnibus profile, glioma, prognosis

## Abstract

High-grade gliomas are the most threatening brain tumors due to aggressive proliferation and poor prognosis. Thus, utilizing genetic glioma biomarkers to forecast prognosis and guide clinical management is crucial. Procollagen-lysine, 2-oxoglutarate 5-dioxygenase 3 (PLOD3) modulates cancer progression and metastasis. However, its detailed function in cancer remains largely uninvestigated. PLOD3 expression was evaluated with real-time PCR in glioblastoma (GBM) cell lines and by Gene Expression Omnibus dataset analysis and immunohistochemistry of glioma tissues. We investigated the clinical use of PLOD3 for determining glioma prognosis. The biological roles of PLOD3 in proliferation, migration and invasion of GBM cells were studied both *in vitro* with wound-healing and transwell assays and *in vivo* using an orthotopic xenograft mouse model. Hypoxia and western blotting were applied to discover the molecular mechanisms underlying PLOD3 functions. PLOD3 mRNA and protein expression were upregulated in glioma tissues compared to normal brain tissues. PLOD3 overexpression was correlated with negative survival in glioma patients. PLOD3 silencing suppressed cell proliferation and induced G1 phase arrest through p53-independent regulation of the p21 pathway. Inhibition of PLOD3 in glioma cells decreased VEGF expression, migration and invasion by downregulating mesenchymal markers, including Snail and Twist. Notably, knockdown of PLOD3 inhibited HIF-1α accumulation via the ERK signaling pathway under hypoxia. Taken together, these discoveries reveal that PLOD3 is a potential therapeutic target in human gliomas.

## INTRODUCTION

Glioblastoma multiforme (GBM) accounts for the most common primary brain tumor occurring among adults, and it responds poorly to current treatments. The median survival time is approximately 14 months, even in patients who undergo aggressive surgical resection and chemo-radiotherapy [1]. The poor prognosis is attributed to invasive features, such as raised mitotic activity and necrosis [2]. The World Health Organization (WHO) outlined the histologic classification of human gliomas [3]. High-grade gliomas have woeful prognosis and negative survival. Despite many genetic markers that are correlated with prognosis, they are not always satisfactory for forecasting prognosis of individual patients [4]. Hence, identification of novel molecular markers is crucial.

Collagen biosynthesis is a complicated process that involves multiple steps and co- and post-translational modifications. Modification of collagen and the extracellular matrix (ECM) is associated with tumor invasion and angiogenesis [5, 6]. Procollagen-lysine, 2-oxoglutarate 5-dioxygenase (PLOD) family members are membrane-bound homodimeric proteins enclosed to the endoplasmic reticulum. PLODs catalyze post-translational lysine residue hydroxylation in collagen-like peptides. These hydroxylysyl groups act as attachment sites for further collagen biosynthesis and are essential for collagen stability [7]. Previous studies have demonstrated that PLODs are not only associated with fibrotic processes and tissue remodeling [8] but they also modulate cancer invasion and metastasis [9–11].

PLOD3, an 85 kDa protein, is one of the three isoforms of the PLOD family [7]. Inactivation of the PLOD3 gene in mouse embryos is lethal, with death around embryonic day 9.5, due to severe insufficiency in type IV collagen synthesis, which causes fragmentation of basement membranes [12]. PLOD3 mutation has been reported in some patients with connective tissue disorders [13]. Recently, PLOD3 overexpression has been found in gastric, colorectal, and pancreatic cancers [14–16]. PLOD3 has also been proposed as a biomarker for radio-resistant human H460 lung cancer stem-like cells [17]. However, its role in determining pathological grading of gliomas and in survival of human glioma patients has not been addressed.

Under the assumption that high-grade brain tumors overexpress PLOD3, we analyzed the Gene Expression Omnibus (GEO) profile dataset and found that PLOD3 expression was positively correlated with WHO grading. Moreover, higher PLOD3 expression was associated with negative survival in glioma patients. These preliminary results suggested that PLOD3 is a potential biomarker for glioma. The wet lab results, including RT-PCR and western blotting, demonstrated overexpression of PLOD3 in human glioma cells. We further investigated the role of PLOD3 in proliferation, migration, and invasion of GBM cells. Finally, our data demonstrated that PLOD3 is a potential prognostic biomarker and therapeutic target in human gliomas.

## RESULTS

### PLOD3 expression is positively associated with WHO pathological grading and negative survival in glioma patients

**Figure 1:**
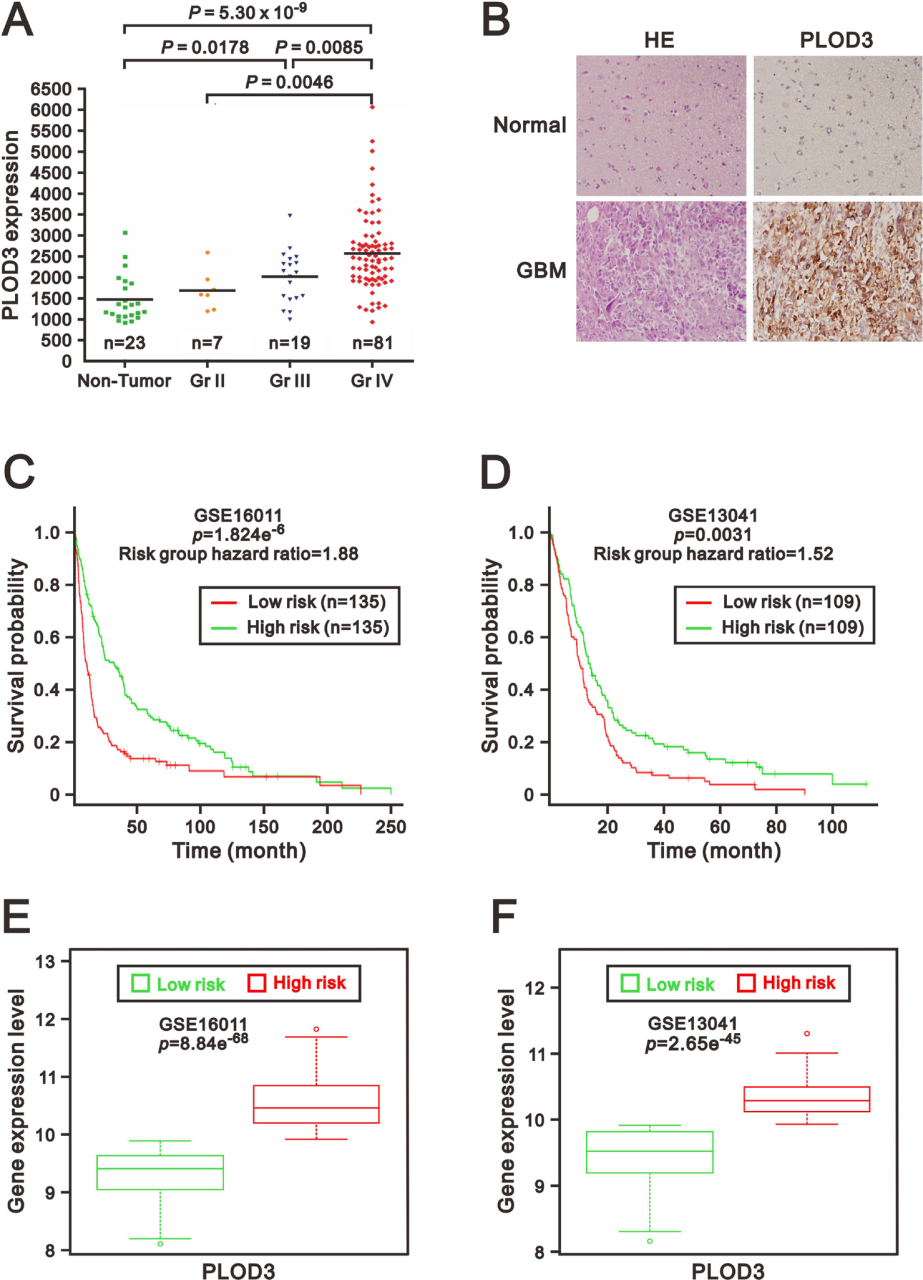
PLOD3 was overexpressed in glioma tissues (**A**) PLOD3 mRNA expression in non-tumor control groups and glioma. The scatter plots show the PLOD3 gene expression in non-tumor controls and in WHO grade II, III and IV gliomas from a GEO dataset (GSE4290). Elevated PLOD3 mRNA levels were positively correlated with WHO grading of gliomas. The adjusted *p* value was calculated between each group. (**B**) Hematoxylin and eosin (HE) staining and immunohistochemical analysis of PLOD3 expression of non-neoplastic brain tissue and glioblastoma multiforme (GBM). The PLOD3 expression in GBM was higher than in non-neoplastic brain tissue. (**C**, **D**) Kaplan–Meier survival curves were generated via the SurvExpress program for investigation of glioma samples from GSE16011 (C) and GSE13041 (D) datasets. Green and red represent low and high expression groups of PLOD3, respectively. The insets in the top right disclose the number of individuals in each risk group. Symbols (+) indicate censored samples. (**E**, **F**) Box plots comparing differences in PLOD3 gene expression in GSE16011 (E) and GSE13041 (F) datasets between risk groups using Student’s *t*-test. Green and red represent low- and high-risk groups, respectively.

To determine PLOD3 expression in glioma tissues, we first analyzed the association between PLOD3 expression and pathological grading of human gliomas using the GEO database (GSE4290). Significantly higher PLOD3 expression was found in WHO pathological grade IV (*n* = 81) than in pathological grade II gliomas (*n* = 7; *p* = 0.0046) and non-tumor controls (*n* = 23; *p* = 5.3 × 10^–9^) (Figure 1A). The PLOD3 level was also elevated in WHO grade III gliomas than in non-neoplastic brain tissues (*n* = 23; *p* = 0.0178). To investigate PLOD3 protein levels in non-neoplastic brain tissues and human GBM, immunohistochemical (IHC) staining of a human tissue microarray was performed (Figure 1B). The PLOD3 expression in GBM was higher than in non-neoplastic brain tissue. Our data established PLOD3 overexpression in high-grade gliomas.

We then used the SurvExpress to examine the correlation between PLOD3 mRNA expression and survival and risk [18]. The survival curves and risk groups were derived from an analysis program on the basis of each glioma's mRNA expression level. Red represents high expression groups, and green represents low expression groups. We surveyed PLOD3 expression in 2 glioma datasets (GSE16011 and GSE13041) from the SurvExpress database [19, 20]. The results from 270 or 218 glioblastoma samples from the GSE16011 and GSE13041 datasets revealed that PLOD3 overexpression was correlated with poor survival (*p* = 1.824e^–6^, *p* = 0.0031, respectively) (Figure 1C and 1D) and was eminently increased in the high-risk group (*p* = 8.84e^−68^, *p* = 2.65e^−45^, respectively) (Figure 1E and 1F). These findings suggest that PLOD3 overexpression in glioma is indicative of a negative prognosis.

### PLOD3 mRNA and protein is overexpressed in human glioma cells

**Figure 2:**
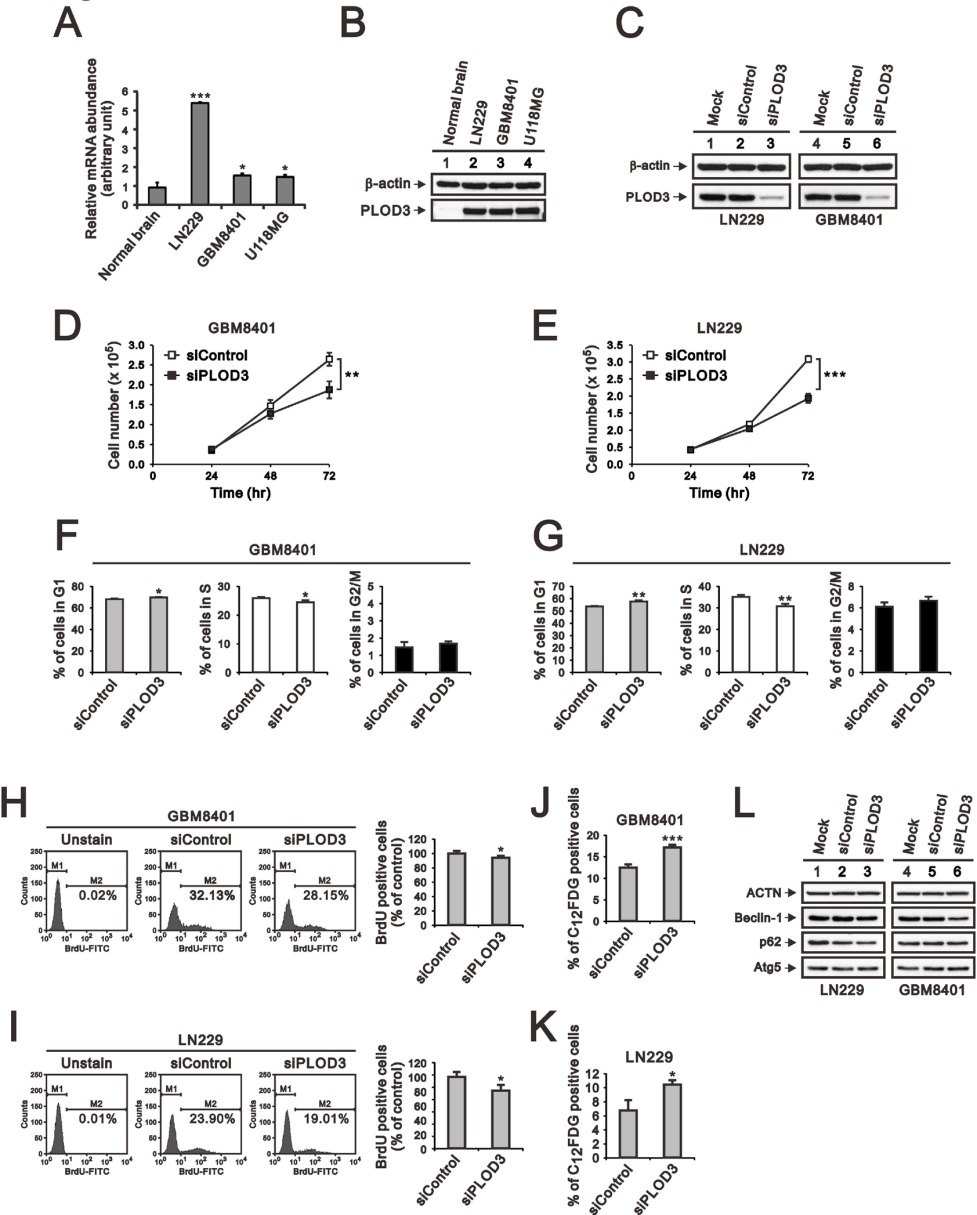
PLOD3 mRNA and protein expression in normal brain tissue and human glioma cell lines (**A**, **B**) Expression of PLOD3 mRNA (**A**) and protein in LN229, GBM8401 and U118MG glioma cell lines (B) and normal brain tissue. The quantitative results of PLOD3 mRNA expression represent data from three independent experiments. The relative gene expression was standardized with that in normal brain. ^*^p < 0.05 and ^***^p < 0.001 compared to the normal brain tissue group. (**C**) Lysates were collected from GBM8401 and LN229 cells 48 h after transfection with 25 nM siRNA. Blots were probed with antibodies targeting total PLOD3. Duplicate biological samples were collected for GBM8401 and LN229 cells. β-actin was used as a loading control. (**D**, **E**) GBM8401 and LN229 cells were transfected with 25 nM siRNA. Cell number was determined at the indicated time points. The data are expressed as the mean ± s.d.; *n* = 3; ^**^p < 0.01, and ^***^p < 0.001. (**F**, **G**) GBM8401 and LN229 cells were stained with propidium iodide (PI) for cell cycle analysis using flow cytometry. The data are expressed as the mean ± s.d.; *n* = 3; ^*^p < 0.05, ^**^p < 0.01. (**H**, **I**) GBM8401 and LN229 cells were labeled with BrdU and then processed for flow cytometric analysis. The data are shown as the means ± s.d.; *n* = 3; ^*^p < 0.05. (**J**, **K**) GBM8401 and LN229 cells were labeled with C_12_FDG for senescence activity assay by flow cytometric method. The data are shown as the mean ± s.d.; *n* = 3; ^*^p < 0.05, and ^***^p < 0.001 compared to the siPLOD3 group. (**L**) Western blot analysis was performed to detect changes in the expression of autophagy associated proteins. The results represent data from two independent experiments. ACTN served as a loading control.

We next analyzed PLOD3 mRNA expression using qRT-PCR in three GBM cell lines, LN229, GBM8401, and U118MG. As shown in Figure 2A, PLOD3 expression was obviously elevated in these GBM cell lines matched against normal brain tissue. We further performed western blotting to quantitate the PLOD3 protein level in human glioma cell lines and normal brain tissue. The PLOD3 protein level was also strongly higher in the three human GBM cell lines (Figure 2B).

### PLOD3 silencing results in a significant inhibition of cell proliferation

To explore the mechanism by which PLOD3 contributes to glioma tumorigenesis, we knocked down PLOD3 expression in GBM8401 and LN229 cells using siRNA. The data showed that siRNA targeting PLOD3 significantly lessened its expression compared with Mock and Control siRNA in GBM8401 and LN229 cells (Figure 2C). PLOD3 has been reported to affect cell proliferation [21]. Thus, we investigated cell proliferation by counting cells. PLOD3 silencing statistically decreased the GBM8401 and LN229 cell numbers (Figure 2D and 2E). Cell cycle analysis showed that PLOD3 knockdown instigated G1 phase arrest (Figure 2F and 2G). Furthermore, we used BrdU incorporation followed by flow cytometry analysis to evaluate cell proliferation. As shown in Figure 2H and 2I, knockdown of PLOD3 decreased the number of active proliferating cells compared with siControl glioma cells. Based on the biological function of PLOD3, it may not alter cell cycle directly. We further investigated autophagy and senescence that may also lower down the cell proliferation in these siPLOD3 glioma cells. There was no obvious change of autophagy markers, including Beclin-1, p62, and Atg5 (Figure 2L). However, there were increased C_12_FDG senescence cells with siPLOD3 than siControl (Figure 2J and 2K). Therefore, the increased senescence cells also contribute to decrease the proliferation of GBM8401 and LN229 glioma cells with siPLOD3. Our results insinuate that PLOD3 may act as an essential character in GBM growth.

### PLOD3 is important for glioma cell migration and invasion

**Figure 3:**
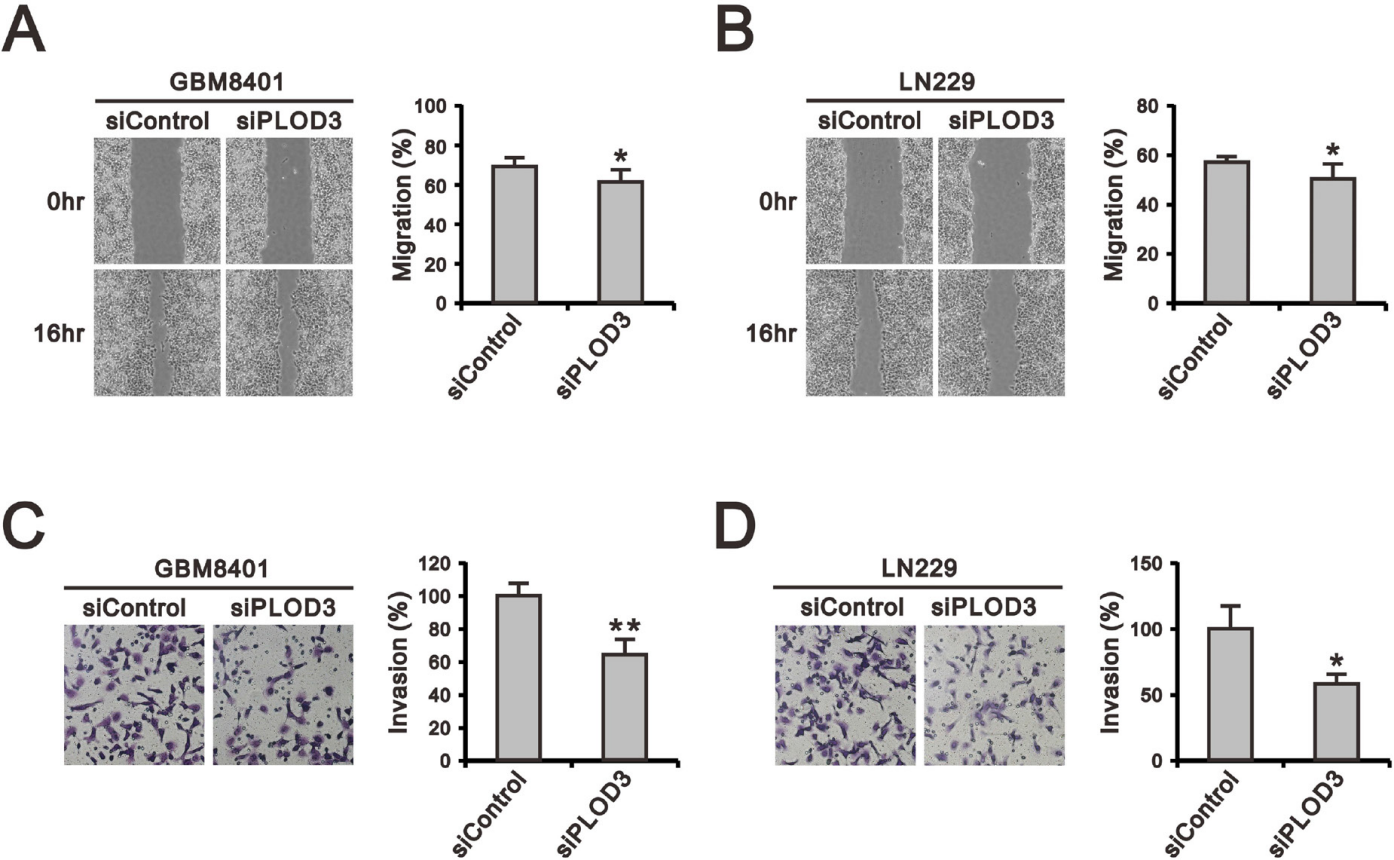
The effect of PLOD3 knockdown on glioma cell migration and invasion (**A**, **B**) Representative images of the wound-healing assay of siPLOD3 compared with siControl GMB8401 (A) and LN229 cells (B). (**C**, **D**) The images show invasion of siControl and siPLOD3 GBM8401 (C) and LN229 cells (D). Histograms show the number of migrating and invading cells. The data are expressed as the mean ± s.d. of triplicate samples from three independent experiments. ^*^p < 0.05 or ^**^p < 0.01 compared to the siControl group.

Overexpression of PLOD isoforms has been demonstrated to act as a critical role in tumor migration via modification of collagen networks in human cancer experiments, but the role of PLOD3 in glioma is poorly studied [11, 22, 23]. Therefore, we performed wound-healing, migration (Figure 3A and 3B) and Matrigel-based invasion assays (Figure 3C and 3D) to investigate the functional role of PLOD3 in GBM8401 and LN229 cell migration and invasion. Our data indicate that PLOD3 silencing significantly reduced invasion and migration of GBM8401 and LN229 cells.

### The effects of PLOD3 silencing on angiogenesis, signaling pathways, and EMT

**Figure 4:**
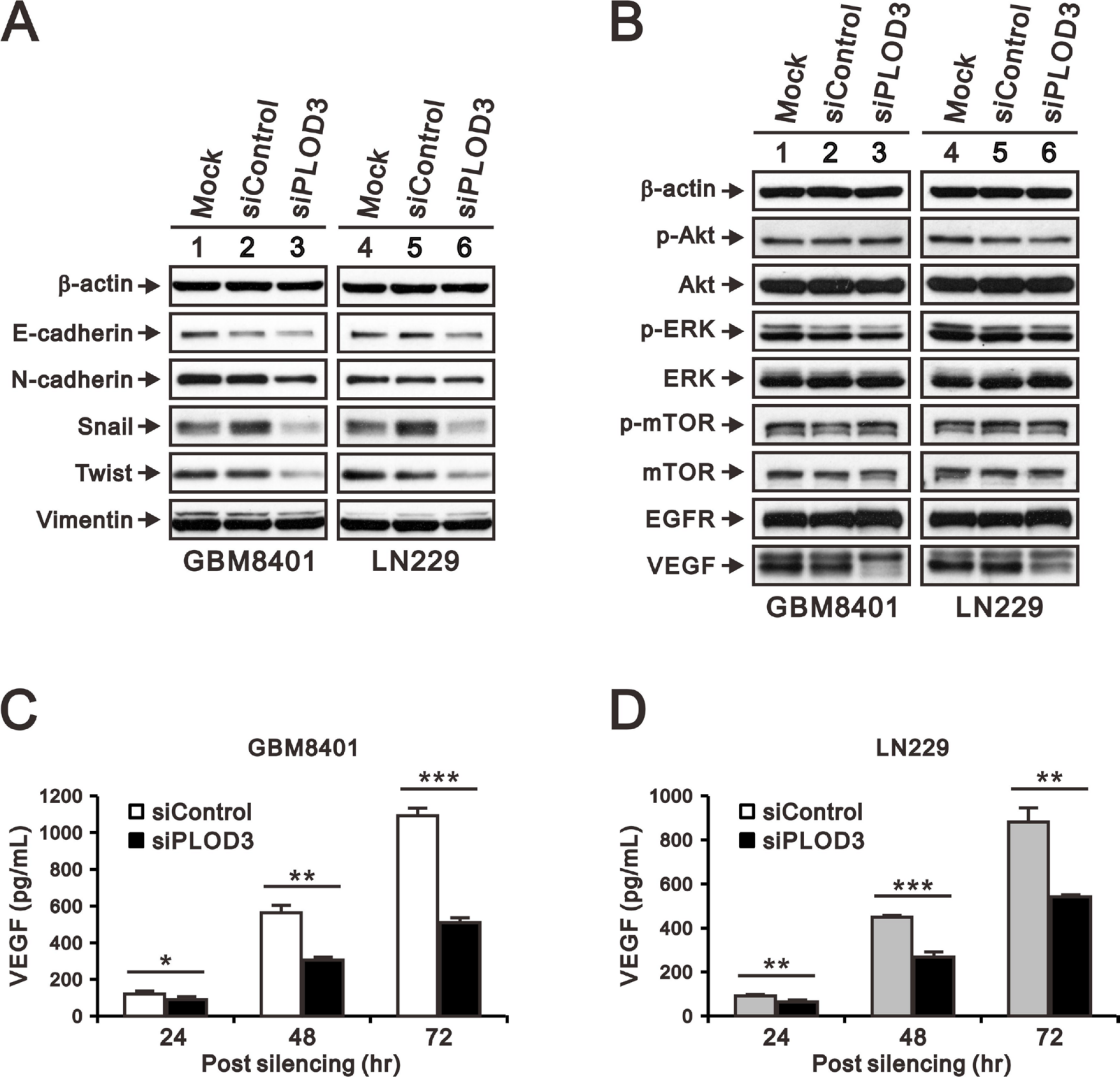
PLOD3 regulates VEGF and EMT marker expression in GBM cells (**A**) Expression of EMT markers (E-cadherin, N-cadherin, Snail, Vimentin, and Twist) in siControl and siPLOD3 GBM8401 and LN229 cells. (**B**) PI3K/AKT/mTOR signaling pathway proteins, including p-AKT (Ser473), total AKT, p-ERK (Thr202/Tyr204), total ERK, p-mTOR (Ser2448), and total mTOR, EGFR, and VEGF were analyzed by western blotting in siControl and siPLOD3 GBM8401 and LN229 cells. The results shown represent data from two independent experiments. β-actin served as a loading control. (**C**, **D**) Protein levels of VEGF in siControl versus siPLOD3 GBM8401 and LN229 cells were measured at 24 h, 48 h, and 72 h after silencing. Data are given as means ± s.d. of at least three independent experiments. ^*^p < 0.05, ^**^p < 0.01, and ^***^p < 0.001 compared to the siControl group.

The phenotypic characteristics of GBM include aggressive invasion, which depends on angiogenesis for rapid growth and pathological progression [24, 25]. Collagens are important ECM components that interact with tumor cells via direct mechanical force or storage of signals. Previous studies have demonstrated that collagen modifying enzyme is associated with angiogenesis and epithelial mesenchymal transition (EMT), which takes an important part in GBM metastasis [6, 22, 26]. Thus far, our results clearly demonstrated that PLOD3 knockdown reduced GBM cell growth and migration. Next, we examined the underlying PLOD3 mechanism in glioma, including EMT markers and the PI3K/AKT/mTOR signaling pathway, which has been reported to play a critical role in regulating angiogenesis in glioma [27, 28]. The VEGF, EGFR, ERK, AKT, mTOR and EMT marker (E-cadherin, N-cadherin, Vimentin, Snail, and Twist) expression in GBM8401 and LN229 cells was measured by western blotting. The western blotting demonstrated that transient PLOD3 silencing reduced Snail and Twist expression (Figure 4A). The signaling pathway protein levels, including AKT, ERK and mTOR were mainly unchanged, except VEGF accumulation decreased after PLOD3 knockdown (Figure 4B). We further performed enzyme-linked immunosorbent assay (ELISA) to check the amount of VEGF secreted by glioma cells in culture medium. siPLOD3 glioma cells significantly had decreased VEGF secretion than control cells at 24 h, 48 h, and 72 h (Figure 4C and 4D). These results indicated that PLOD3 may affect angiogenesis. In addition, PLOD3 altered EMT by regulating Snail and Twist expression.

### Stable PLOD3 knockdown suppresses cell proliferation and clonogenic formation

**Figure 5:**
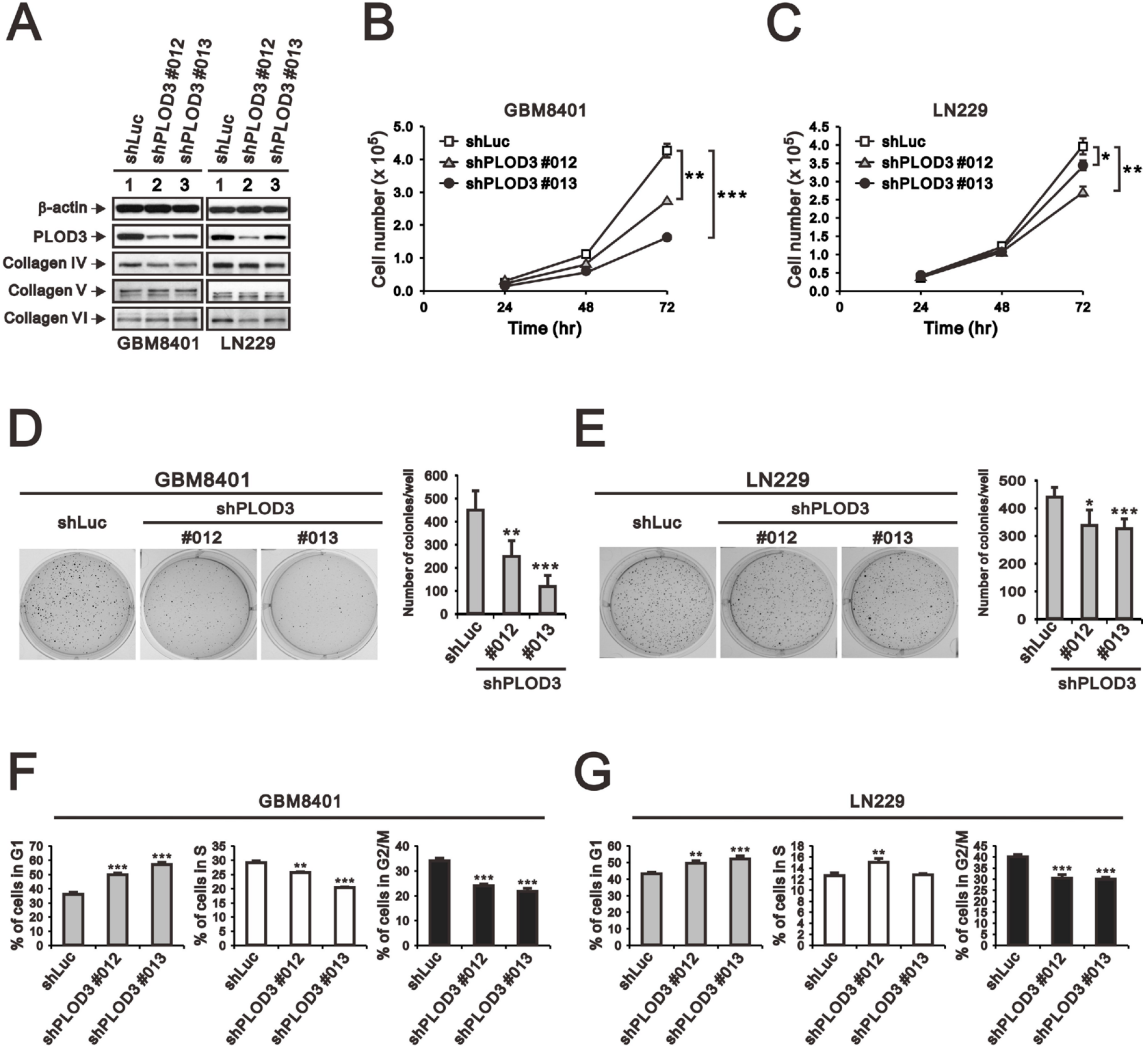
The effect of PLOD3 knockdown on glioma cell proliferation and cell cycle (**A**) SDS-PAGE and western blot analysis of GBM8401 shPLOD3 (#012 and #013), LN229 shPLOD3 (#012 and #013), and shLuc control cells was applied to quantitate PLOD3 and type IV, V, and VI collagen protein expression. β-actin served as a loading control. The protein levels shown represent data from two independent experiments. (**B**, **C**) The number of GBM8401 (**B**) and LN229 (**C**) clones were measured at the indicated time points. The data are expressed as the mean ± s.d.; *n* ≥ 4; ^*^p < 0.05, ^**^p < 0.01, and ^***^p < 0.001. (**D**, **E**) Colony formation analysis of controls and PLOD3-silenced GBM8401 (**D**) and LN229 (**E**) cells was performed. Quantitative analysis of colonies is presented as the mean ± s.d. of at least four independent experiments; ^*^p < 0.05, ^**^p < 0.01, and ^***^p < 0.001. (**F**, **G**) Cell cycle analysis of GBM8401 (**F**) and LN229 (**G**) cells was performed using flow cytometry. Quantitative analysis of colonies is presented as the mean ± s.d. of at least three independent experiments; ^**^p < 0.01, or ^***^p < 0.001 compared to the control shLuc group.

To examine the long-term effect of PLOD3 on glioma cell growth *in vitro* and *in vivo*, stable PLOD3-silenced GBM8401 and LN229 clones were prepared using a lentiviral shRNA system. We evaluated the knockdown effects of four shPLOD3 clones from the RNAi core facility on PLOD3 expression in GBM8401 and LN229 cells (data not shown). shPLOD3#012 and #013 efficiently silenced PLOD3 protein expression in comparison to the shLuc control. Because PLOD3 is a collagen-modifying enzyme, we were interested in knowing whether PLOD3 knockdown would influence type IV, V, and VI collagen levels in glioma cells (Figure 5A). Our data suggest that type IV, V, and VI collagen expression was largely unchanged after PLOD3 knockdown in glioma cells.

After successful generation of stable PLOD3 knockdown glioma cells, we investigated cell proliferation in GBM cells. Cell counting showed a significantly decreased number of GBM8401 and LN229 shPLOD3 cells compared with shLuc control cells (Figure 5B and 5C). A colony-forming assay was performed to survey the role of PLOD3 on cell survival. The number of GBM8401 and LN229 shPLOD3 cell colonies was statistically decreased compared with that of the control cells (Figure 5D and 5E). We also performed a cell cycle assay and found that both GBM8401 and LN229 shPLOD3 clones had a significantly higher percentage of G1 phase arrest cells than shLuc glioma cells (Figure 5F and 5G).

To determine whether PLOD3 silencing also caused cell death, apoptosis was determined using Annexin V and 7-Aminoactinomycin (7-AAD) staining followed by flow cytometric analysis. shLuc control or shPLOD3 cells exhibited a similar low (4–6%) degree of cell death (data not shown). Collectively, these results suggested that the decline in cell growth seen upon PLOD3 knockdown is due to repression of cell proliferation and not enhancement of cell death.

### PLOD3 silencing decreases tumor mass in a mouse xenograft animal model

**Figure 6:**
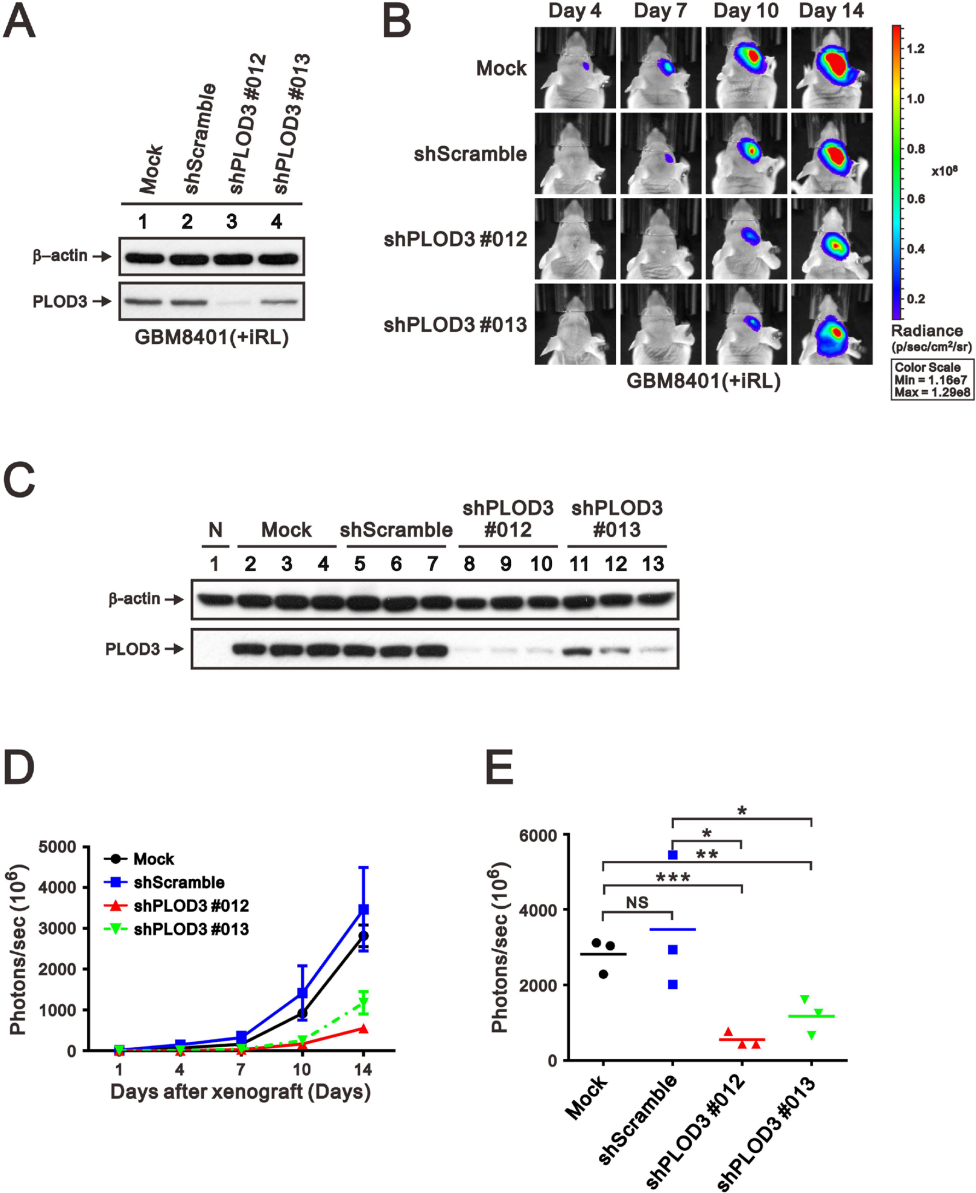
PLOD3 knockdown decreased glioma growth in an orthotopic mouse model (**A**) SDS-PAGE and western blot analysis of GBM8401-iRL cells transfected with shScramble and shPLOD3 RNA were applied to quantitate PLOD3 protein expression. (**B**) Representative IVIS images of mice (days 4, 7, 10, and 14) after implantation of GBM8401-iRL cells stably transfected with shScramble or shPLOD3 RNA. (**C**) Western blot analysis was carried out of normal brain, and tumors formed by Mock, shScramble, shPLOD3#012, and shPLOD3#013 GBM8401-iRL cells. Tumor growth curve (**D**) and quantitative analyses of tumor progression at day 14 (**E**) based on the total intensity of IVIS imaging. *n* = 3 for each group; ^*^p < 0.05, ^**^p < 0.01, and ^***^p < 0.001 compared to the PLOD3 wild-type group.

To evaluate the anti-cancer effect of PLOD3 silencing, a GBM8401-iRL cell line with stable PLOD3 knockdown was generated and applied to a xenograft mouse model (Figure 6A). After 2 weeks of tumor cell transplantation, the PLOD3 knockdown glioma cells developed significantly smaller tumors compared with PLOD3 wild-type cells (Figure 6B). The expression of PLOD3 in tumors were examined by western blot. There are significantly decreased PLOD3 abundance in shPLOD3 GBM8401-iRL groups than Mock and shScramble groups (Figure 6C). Tumor growth statistically decreased with PLOD3 silencing compared with the GBM8401-iRL mock and shScramble group (Figure 6D and 6E). These results show that knockdown of PLOD3 repressed glioma cell growth *in vivo*.

### PLOD3 downregulate hypoxia triggered HIF-1α expression in glioma cells

**Figure 7:**
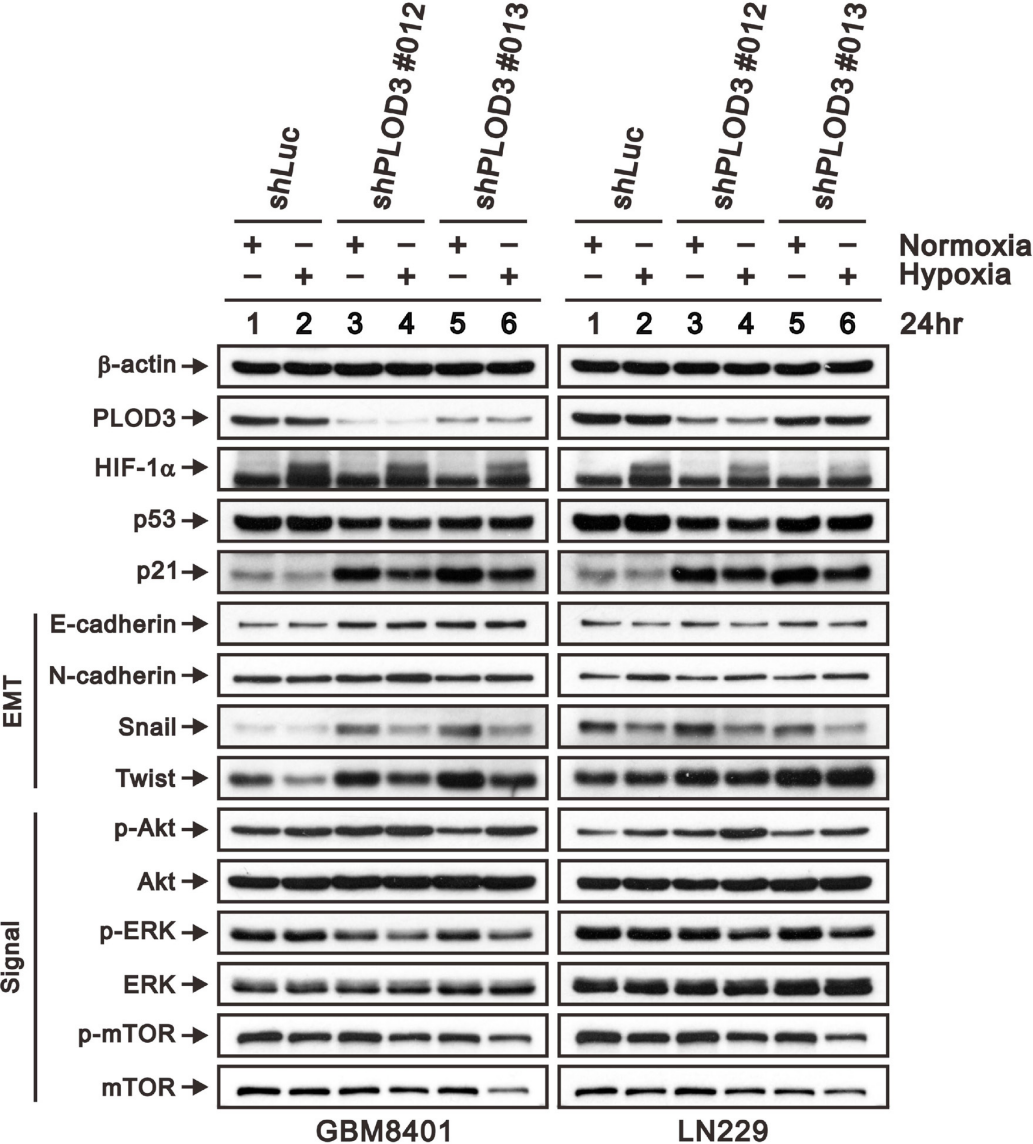
PLOD3 regulates p21 and hypoxia induced HIF-1α expression in GBM cells Expression of HIF-1α, p53, p21, EMT markers (E-cadherin, N-cadherin, Snail, and Twist), and PI3K/AKT/mTOR signaling pathway proteins (p-AKT (Ser473), total AKT, p-ERK (Thr202/Tyr204), total ERK, p-mTOR (Ser2448), and total mTOR) was analyzed by western blotting in shLuc, shPLOD3 GBM8401, and LN229 cells under normoxic and hypoxic conditions. The data are representative of two independent experiments. β-actin served as a loading control.

Hypoxia or HIF-1 overexpression has been reported to induce glioma proliferation, migration and invasion [29]. Hofbauer’s study found that PLODs are triggered by hypoxia via the hypoxia-inducible factor 1 (HIF-1) pathway [30]. To determine the association between PLOD3 and hypoxia or the HIF-1 pathway, we cultured GBM8401 and LN229 cells in hypoxic chamber for 24 h, and the PLOD3 expression was evaluated via western blot analysis. Because our cell cycle studies revealed that PLOD3 silencing results in G1-phase arrest of GBM8401 and LN229 cells, we examined the effect of PLOD3 knockdown on cell cycle-regulatory molecules functioning in the G1 phase. We evaluated the effect of PLOD3 silencing on induction of p21 and p53, which are known to control the access of cells at the G1-S-phase transition checkpoint. Moreover, we also investigated ERK, AKT, and mTOR expression in GBM8401 and LN229 cells in normoxic and hypoxic environments. As shown in Figure 7, western blotting demonstrated that PLOD3 silencing resulted in elevated E-cadherin, Snail, Twist, p-AKT and p21 expression without p53 accumulation. Interestingly, HIF-1α, Snail, Twist and p-ERK expression was more downregulated in shPLOD3 glioma cells compared with shLuc control cells under hypoxic conditions than under normoxic conditions. The levels of mTOR and p-mTOR were largely unchanged. These results indicated that the anti-tumorigenicity of PLOD3 is associated with the p53-independent p21 pathway in glioma. In addition, PLOD3 plays a positive role in HIF-1α accumulation and phosphorylation of ERK pathway proteins under hypoxia. Furthermore, PLOD3 regulates the expression of hypoxia-induced EMT markers, including E-cadherin, Snail and Twist.

## DISCUSSION

GBM is the most frequent type of adult primary brain tumor and well known for its short survival time. Rapid growth and aggressive invasion of GBM contribute to practically impossible to completely resect the tumor and high recurrence [1]. Therefore, a combination of radiation and chemotherapy with temozolomide, an alkylating agent, is necessary as standard treatment after surgery [31]. However, tumor cells with O-6-methylguanine-DNA methyltransferase (MGMT) gene expression are capable of repairing the DNA damage induced by temozolomide [32]. Thus, other therapeutic agents for malignant glioma are highly desired [33, 34].

PLODs are a family of intracellular membrane-bound homodimeric proteins. Notably, PLOD3 not only has lysyl hydroxylase activity similar to PLOD1 and PLOD2 but also possesses collagen galactosyltransferase [35] and glucosyltransferase [36, 37] activities *in vitro* and *in vivo* [21, 38]. Feres-Filho *et al.* has reported that the C-terminal region of PLOD3 is responsible for the lysyl hydroxylase activity, whereas the N-terminal region is accountable for glycosyltransferase activity [39]. Wang *et al.* has demonstrated that PLOD3 silencing induced a decline in proliferation and viability of fibrosarcoma HT-1080 cells [21]. Moreover, PLOD3 overexpression is found in gastric, colorectal, and pancreatic cancers [14–16]. These studies provided evidence that PLOD3 is associated with tumor progression. Our data confirm that elevated PLOD3 mRNA expression is associated with high-grade gliomas and unfavorable prognosis in two different datasets, GSE16011and GSE13041 [19, 20]. These results provide evidence that PLOD3 may be an oncogene associated with tumor malignancy in glioma patients.

### PLOD3 inhibited glioma growth via the p53-independent p21 pathway

Aggressive invasion of glioma is highly associated with the ECM. The tumor microenvironment acts an important role to trigger glioma's invasive feature [40, 41]. To analyze the putative pathological role of PLOD3 in glioblastoma, we performed PLOD3 loss-of-function studies. PLOD3 silencing significantly attenuated cell growth and colony formation through cell cycle interruption. Moreover, we found that PLOD3 knockdown increased G1 arrest by increasing the expression of p21 without p53 accumulation. p21 is a cell cycle inhibitor and largely regulated by the tumor-suppressor protein p53, which is a crucial player in maintaining the equilibrium between cell growth and cell death. p53 mutation prevents growth arrest of damaged cells by regulating cell cycle checkpoints that promote tumorigenesis in various human cancers, including GBM [42, 43]. However, p21 upregulation helps maintain genomic integrity by inhibiting the cyclin-dependent kinase CDK1 that interrupts G1 phase to S phase transition, aiding the repair of injured DNA [44]. Thus, our study indicates that PLOD3 silencing may have anti-proliferative effects in the deficiency of active (wild-type) p53 through p21 activation. Further studies are needed to clarify this.

HIF-1α takes an vital part in regulation of stress-responsive genes. Besides, hypoxia is an important inducer for aggressive form of GBM [45, 46]. HIF-1 signaling is not only sensitive to hypoxia but also regulated by growth factors and molecules in ECM [47]. ECM is mainly composed of collagens which give biochemical and biomechanical support to maintain cell function [48]. HIF-1α upregulates a variety of downstream signaling pathways associated with cell survival, motility, and metabolism [49]. Tumor aggressiveness stimulated by HIF-1α has been found to rely on collagen remodeling enzymes, including PLOD2, and the prolyl hydroxylases, but PLOD3 is poorly studied [9, 50]. In this study, we observed that GBM cells with PLOD3 knockdown reduced HIF-1α and phospho-ERK levels but did not affect total ERK protein levels under hypoxic condition. A previous study revealed that the transcriptional response of HIF-1α under hypoxia depends on the ERK pathway [51]. Furthermore, PLOD catalyze post-translational lysine residue hydroxylation in collagen-like peptides which are responsible for collagen biosynthesis and are critical for collagen stability. The PLOD3 silencing may alter ECM property via collagen cross-linking and thus affect the expression of HIF-1α. Taken together, our data indicated that PLOD3 may have an important role in hypoxia-induced HIF-1α expression via the ERK pathway. More studies are needed to clarify the role of PLOD3 in the ERK pathway.

Previous studies have demonstrated that increased ECM stiffness is correlated with elevation of ECM components, including laminin, collagen, and fibronectin, and can promote brain tumor proliferation, invasion and angiogenesis [6, 40, 41]. GBMs exhibit rapid cell proliferation and insufficient vascularization commonly introducing tumor areas with unsatisfactory oxygen supply [45]. This continuing exposure to tremendously low levels of oxygen frequently yields necrotic zones enclosed by densely aggregated hypoxic glioma cells, also named as pseudopalisading GBM cells [52]. Therefore, hypoxia happens in GBM. These pseudopalisading GBM cells are found to upregulate hundreds of hypoxia-associated genes, including cell survival and angiogenesis [45, 52]. Hence, angiogenesis is an important pathological hallmark of GBM, and these GBM cells become more invasive and correlated with poor prognosis [24, 25, 53]. There is a significantly higher level of VEGF mRNA in the pseudopalisading cells surrounding necrotic foci in GBM [54]. VEGF not only promotes endothelial cell proliferation but also boosts endothelial cell migration and invasion [55]. The elaborate glomeruloid microvascular proliferation in GBM is thought to be associated with VEGF overproduction around areas of pseudopalisading necrosis [56]. In contrast, suppression of VEGF expression inhibits angiogenesis and tumor growth, and this effect has been applied in a clinical trial [57]. However, resistance to VEGF inhibition therapy limits the therapeutic benefit [58]. Modifying the collagen matrix through inhibition of the collagen crosslinking enzyme lysyl oxidase alters the tumor microenvironment and decreases angiogenesis and progression of brain tumors *in vitro* and *in vivo* [6]. Our study results showed that PLOD3 silencing also inhibits VEGF expression. The anti-angiogenesis effect of PLOD3 may depend on disarrangement of collagen alignment in glioma, which has been observed in an *in vivo* xenograft study with knockdown of PLOD2, the other PLOD3 isoform [22]. Combining anti-PLOD3 therapy with radiation or temozolomide may be a potential therapeutic strategy.

Growing evidence has shown that tumor cells will produce secretions under stress, such as hypoxia, chemotherapy, and radiotherapy, that alter their microenvironment to enable tumor angiogenesis and metastasis [59]. These extracellular tumor signals will change the extracellular matrix to allow their adjustment to stress [60]. Aberrant EMT activation is a critical mechanism for glioma progression [26]. Typical EMT characteristics include modifications in gene expression, lack of cell-cell contacts, and acquirement of migratory and invasive capability associated with metastatic ability [61]. Decreased E-cadherin expression and increased expression of mesenchymal markers, such as Snail and Twist, are markers of EMT [61]. EMT could be categorized into three general subtypes [62]. Snail and Twist are transcriptional factors and associated with EMT type 1 to 3, but vimentin belongs to cytoskeletal marker and associated with EMT type 1 and 2. Type 3 EMT takes a part in the multiple-step process of metastasis. Our study result demonstrated that PLOD3 silencing inhibits glioma cell migration that could be categorized as type 3 EMT due to suppressing Snail and Twist expression, but no change of Vimentin.

In this study, there are inconsistent expression of EMT markers between glioma cells treated with siPLOD3 and shPLOD3 which may be explained by the cells’ response to acute and long-term knockdown of PLOD3. Both small interfering RNA (siRNA) and shRNA (short hairpin RNA) are the tools mediated gene silencing. However, siRNA and shRNA are fundamentally different molecules [63]. Theoretically, the silencing effect of shRNA should be a permanent condition. Thus, siRNA has short-term and shRNA has long-term gene silencing effect, respectively. The long-term PLOD3 knockdown may change collagen cross-linking and further modify ECM. Therefore, the expression of EMT markers may be different between siRNA and shRNA

Collagens are vital constituent of the ECM that affect tumor malignant features through collagen receptors, such as integrins and Endo180 [48]. Increased expression of collagen type IV and VI and collagen modifying enzymes is observed in brain tumors [6]. Our data showed that PLOD3 silencing did not alter the amount of collagen IV, V, or VI. Previous glioma studies that manipulated collagen-modifying enzymes, including PLOD2 and LOX knockdown, demonstrated similar results [6, 22]. However, decreased collagen crosslinking after PLOD knockdown in tumor stroma has been reported [6, 23]. The Chen *et al.* reported there are no macromolecular and ultrastructural difference of collagens between PLOD2-deficient and control tumors [23]. However, PLOD2-deficient tumors had less stiffness which suggested that PLOD2 is an essential factor for collagen cross-links and associated with the biomechanical property of tumor [23]. PLOD3 may also have impact on collagen cross-links that alter the migratory and invasive properties of GBM cells. Moreover, we used 2-dimensional cell culture without ECM components in this study, but real tumors grow in a 3-dimensional environment and interact with various cell types, collagens and protein ligands in the ECM. Therefore, we designed an orthotopic xenograft *in vivo* study to investigate the role of PLOD3 in glioma invasion. In the *in vitro* study, PLOD3 knockdown suppressed tumor growth and progression. Experiments to examine the role of PLOD3 in cytoskeletal changes, matrix stiffness and ECM composition merit further study.

## CONCLUSIONS

In summary, we report here a previously unrecognized pathological role for deregulated PLOD3 in human glioblastoma. The expression of PLOD3 is a noteworthy biomarker for human glioma prognosis. This enzyme is also involved in glioma cell growth and invasiveness *in vitro* and *in vivo*. We hope this PLOD3 study provides valuable insight into novel therapeutic stratagems for glioma.

## MATERIALS AND METHODS

### Cell culture, siRNA and shRNA transfection

LN229 and GBM8401 cells were harvested in Dulbecco’s modified Eagle’s medium (DMEM) containing 2% fetal bovine serum (FBS), penicillin, and streptomycin at 37° C and 5% CO_2_. U118MG were harvested in DMEM containing 10% FBS, penicillin, and streptomycin at 37° C and 5% CO_2_. GBM8401-iRL cells were harvested in RPMI 1640 Medium containing 10% FBS, penicillin, and streptomycin at 37° C and 5% CO2. GBM8401 or LN229 cells were transfected with PLOD3 small interfering RNA (siRNA) (siGENOME SMARTpool, Dharmacon) using the DharmaFECT 1 Transfection Reagent (GE Healthcare Dharmacon Inc.) according to the manufacturer’s instructions. GBM8401-iRL cells were obtained by stable transfection with pLuc2-iRFP and selected with a FACSAria Fusion Sorter. For PLOD3 long-term knockdown experiments, GBM8401 or LN229 cells were infected with shLuc control and PLOD3-shRNA-containing lentiviral vectors (Academia Sinica, Taiwan, ROC) over 24 h in the presence of Polybrene, followed by selection in medium encompassing puromycin (2 g/ml) for 7 days.

### PLOD3 expression, survival analysis, and risk assessment in human glioma

We recruited GSE4290, acquired from http://www.ncbi.nlm.nih.gov/geo/tools/profileGraph.cgi?ID=GDS1962:202185_at, to investigate PLOD3 expression in each glioma grade. The procedural details have been previously described [64]. We used the online biomarker tool SurvExpress to investigate the survival analysis and risk assessment of PLOD3 in two human glioma datasets (GSE16011; GSE13041) [18]. The glioma patients were categorized into high- and low-risk groups on the basis of each participant's genetic profiles and survival by Cox regression analysis.

### RNA isolation and quantitative real-time reverse transcription-PCR

We performed total mRNA extraction, reverse transcription and quantitative RT-PCR according to the manufacturer’s protocol. Normal brain cDNA was purchased from Origene Technologies (Rockville, MD, USA). The reverse transcripts were amplified and quantified using an Illumina ECO™ Real-Time PCR system. The relative quantitative gene expression was normalized to GAPDH as an internal control and calculated using the 2^−ΔΔ*C*t^ method. The primer pairs used were as follows: PLOD3 forward 5′-GACCCGGTCAACCCAGAGA-3′ and reverse 5′-CTCCACCAACTGTTCGAGCC-3′ [65]; GADPH forward 5′-CTTCATTGACCTCAACTAC-3′ and reverse 5′-GCCATCCACAGTCTTCTG-3′.

### Cell lysate preparation and western blot analysis

Cells were homogenized in lysis buffer (100 mM Tris-HCl, 150 mM NaCl, 0.1% SDS, and 1% Triton-X-100) at 4° C and separated via SDS-PAGE electrophoresis. Protein lysates of normal brain tissue were purchased from Abcam. The proteins were then electrophoretically transferred onto a polyvinylidene difluoride membrane (Bio-Rad Laboratories, Inc.). Primary antibodies specific for PLOD3 (Sigma-Aldrich), Beclin-1 (Cell Signaling Technology), p62 (Santa Cruz Biotechnology), Atg-5 (Cell Signaling Technology), E-cadherin (Cell Signaling Technology), N-cadherin (Cell Signaling Technology), Vimentin (Cell Signaling Technology), Snail (Cell Signaling Technology), Twist (Santa Cruz Biotechnology), p-AKT (Ser473) (Cell Signaling Technology), AKT (Abcam), p-ERK (Thr202/Tyr204), ERK (Cell Signaling Technology), p-mTOR (Ser2448), mTOR (Cell Signaling Technology), EGFR (Cell Signaling Technology), VEGF (Santa Cruz Biotechnology), HIF-1α (Santa Cruz Biotechnology), p21 (Santa Cruz Biotechnology), p53 (Santa Cruz Biotechnology), ACTN (Santa Cruz Biotechnology), and β-actin (Sigma-Aldrich) and Collagens IV, V and VI (Abcam) were used.

### Enzyme-linked immunosorbent assay (ELISA)

The levels of VEGF in the culture medium from two glioblastoma cell lines (GBM8401 and LN229) were measured using an ELISA kit (R&D Systems, Inc., Minneapolis, MN) at 24, 48, and 72 hours.

### Immunohistochemistry

A human glioma tissue microarray was purchased from US Biomax (BS17015a) and immunostained with anti-PLOD3 antibody and stained with hematoxylin and eosin (Sigma-Aldrich, St. Louis, MO, USA).

### Cell proliferation, soft agar colony formation assays and flow cytometry cell cycle analysis

For cell counting assay, GBM8401 and LN229 cells (5 × 10^4^) were seeded per well of a 12-well plate. The cells were counted each day to detect differences in growth rate between the experimental groups and the control groups in five independent experiments. For colony formation in soft agar, we seeded GBM8401 and LN229 cells (2 × 10^3^) in each well of six-well plates with medium containing 0.35% SeaPlaque Agarose (Lonza Rockland, Inc.) on top of a base medium containing 0.5% agarose. Colonies were stained 2 weeks later with crystal violet, and only colonies more than 0.05 mm were numbered using ImageJ software (NIH, Bethesda, MD). For cell cycle analysis, the cells in each group were fixed in 70% ethanol at 4° C and kept at –20° C overnight. The GBM8401 and LN229 cells were then washed twice with cold phosphate-buffered saline (PBS), and stained with propidium iodide (PI) solution (50 μg/ml PI in PBS, 1% Tween 20 and 10 μg/ml RNase A) for 30 min in the dark. Cell-cycle analysis was performed by measuring DNA content using fluorescence activated cell sorting (BD Biosciences, San Jose, CA, USA). Two independent experiments were performed. For the proliferation assay, the glioma cells were processed with the FITC-BrdU Flow Kits according to the manufacturer’s instructions (BD Biosciences), and measured by flow cytometry.

For senescence activity assay by flow cytometry, cells were stained with C_12_FDG, a substrate producing fluorescence and membrane impermeable when cleaved by β-galactosidase. Glioma cells were trypsinized with trypsine EDTA, washed with PBS, resuspended in 50 µl PBS with C_12_FDG for 10 minutes. Then, glioma cells were analyzed immediately using a FACS Calibur flow cytometer, and data were analyzed by FACSDiva software (BD Biosciences, San Jose, CA, USA). The fluorescein signal of C_12_FDG was measured on the FL1 detector. β-galactosidase activity was evaluated as the median fluorescence intensity (MFI) of the glioma population.

### Cell migration and invasion assays

The cells in each group were seeded into 12-well plates and grown at 37° C in a 5% CO_2_ incubator. We removed the medium when cell confluence reached 90% and made a wound in the monolayer with a pipette tip. Then, we washed the plate for three times to expel the non-adherent cells. The wound area was photographed immediately after wounding (0 h) and at 16 h post-wounding. The migration rates were computed according to the change of wound area measured by ImageJ software (NIH, Bethesda, MD).

Transwell chambers (BD Biosciences, San Jose, CA) were used for the invasion study. Glioma cells in serum-free DMEM were added to the upper chambers, which were coated with a thin layer of Matrigel matrix, and medium containing 5% FBS was added to the lower chambers. The cells were incubated at 37° C in a CO_2_ incubator for 16 h. After removing the non-migrated cells, the upper chambers were stained with 0.1% crystal violet for 10 mins, and cells were counted under a microscope.

### Orthotopic tumor xenografts in nude mice

All experimental protocols were approved by the Institutional Animal Care and Use Committee (IACUC) of the National Defense Medical Center, Taiwan. Briefly, 5- to 6-week-old nude female mice (National Laboratory Animal Center, Taiwan) were placed in a stereotactic frame after anesthetization.We made a midline scalp incision, and then drilled a burr hole in the right side of the skull (2.5 mm lateral to the midline and 0.4 mm posterior to the bregma). We injected 2.5 × 10^5^ GBM8401-iRL cells to the target site, depth of 3 mm from the brain surface, by a 10 μl Hamilton syringe. At 2 weeks after transplantation, all the mice were killed. Tumor growth was monitored with bioluminescence imaging using IVIS on days 4, 7, 10, and 14.

### Statistical analysis

Student’s *t*-test or one-way analysis of variance was used for comparisons between groups. The results are presented as the means ± s.d. or as specified. ^*^p < 0.05, ^**^p < 0.01, and ^***^p < 0.001 were considered to indicate significant differences.

## Abbreviations

PLOD3: Procollagen-lysine, 2-oxoglutarate 5-dioxygenase 3
GBM: glioblastoma multiforme
ECM: extracellular matrix
GEO: Gene Expression Omnibus
WHO: World Health Organization
IHC: immunohistochemical
EMT: epithelial mesenchymal transition
ELISA: enzyme-linked immunosorbent assay
